# Image Analysis and Computer Vision Applications in Animal Sciences: An Overview

**DOI:** 10.3389/fvets.2020.551269

**Published:** 2020-10-21

**Authors:** Arthur Francisco Araújo Fernandes, João Ricardo Rebouças Dórea, Guilherme Jordão de Magalhães Rosa

**Affiliations:** ^1^Department of Animal and Dairy Sciences, University of Wisconsin-Madison, Madison, WI, United States; ^2^Department of Biostatistics and Medical Informatics, University of Wisconsin-Madison, Madison, WI, United States

**Keywords:** computer vision, sensors, imaging, phenotyping, automation, livestock, precision livestock, high-throughput phenotyping

## Abstract

Computer Vision, Digital Image Processing, and Digital Image Analysis can be viewed as an amalgam of terms that very often are used to describe similar processes. Most of this confusion arises because these are interconnected fields that emerged with the development of digital image acquisition. Thus, there is a need to understand the connection between these fields, how a digital image is formed, and the differences regarding the many sensors available, each best suited for different applications. From the advent of the charge-coupled devices demarking the birth of digital imaging, the field has advanced quite fast. Sensors have evolved from grayscale to color with increasingly higher resolution and better performance. Also, many other sensors have appeared, such as infrared cameras, stereo imaging, time of flight sensors, satellite, and hyperspectral imaging. There are also images generated by other signals, such as sound (ultrasound scanners and sonars) and radiation (standard x-ray and computed tomography), which are widely used to produce medical images. In animal and veterinary sciences, these sensors have been used in many applications, mostly under experimental conditions and with just some applications yet developed on commercial farms. Such applications can range from the assessment of beef cuts composition to live animal identification, tracking, behavior monitoring, and measurement of phenotypes of interest, such as body weight, condition score, and gait. Computer vision systems (CVS) have the potential to be used in precision livestock farming and high-throughput phenotyping applications. We believe that the constant measurement of traits through CVS can reduce management costs and optimize decision-making in livestock operations, in addition to opening new possibilities in selective breeding. Applications of CSV are currently a growing research area and there are already commercial products available. However, there are still challenges that demand research for the successful development of autonomous solutions capable of delivering critical information. This review intends to present significant developments that have been made in CVS applications in animal and veterinary sciences and to highlight areas in which further research is still needed before full deployment of CVS in breeding programs and commercial farms.

## Introduction

Sighted animals, including humans, experience vision in a way that seems natural and automatic. Early in life, and quite often from the moment of birth, animals use their vision system to navigate the world around them, and to identify and interact with other animals, as well as their surrounding environment. Therefore, the vision system of an animal is constantly being trained and adapted so that it can be used for several tasks. For instance, in humans, this system works with the luminous signal being captured by the eye and transferred via the optic nerve to the brain, where it is processed and interpreted (1). This complex vision system can adapt to different light conditions autonomously while allowing us to focus on objects and to have a 3-dimensional representation of the world. But what would be vision for a computer and how can computer vision impact animal breeding and production? This review is divided into three sections. The first section provides a brief introduction to image analysis and computer vision, describing current developments and algorithms of interest. The second section describes common types of sensors available and their functionality. The third presents a historical view on applications in animal sciences, followed by examples and areas of current interest. The review closes with a discussion on areas that are currently of great importance for the improvement of computer vision system (CVS) applications in livestock improvement and production.

## Overview of Strategies to Work With Digital Images

### What Is Digital Image Processing, Image Analysis, and Computer Vision?

Digital Image Processing, Digital Image Analysis, and Computer Vision can be viewed as an amalgam of terms that very often are used to describe similar processes and applications, generating confusion regarding their meaning. Most of the confusion arises because these are interconnected fields that emerged with the development of technologies for digital image acquisition. For the sake of clarity, we can divide and define these three areas as follows.

#### Digital Image Processing

Digital Image Processing deals with capturing and translating a visual signal into a digital image. As such, it can be viewed as the area that studies the process of obtaining a visual signal of the world and transforming it in order to make it interpretable. It spans from the study of image formation, as a result of the acquisition of light signals by specifically designed sensors, to the interpretation of the image as an array of connected values. Therefore, digital image processing involves the conception, design, development, and enhancement of digital imaging algorithms and programs (2). As such, it is a discipline heavily based on physics and mathematics. The term can also be used to directly address the applications or techniques used for digital image manipulation, ranging from noise reduction, image equalization, image filtering, and other transformations used for preparing images for subsequent steps in an analysis pipeline or for enhancing images aesthetically. A group of techniques of great importance in digital image processing is edge and contour detection. Although there are several methods for edge detection, they all rely on the fact that edges are regions in an image where there is a drastic change in color/intensity along with a particular orientation (2, 3). These techniques are, in general, useful in many applications in image processing, such as image correction and sharpening (i.e., highlight of the edges) and in image analysis, such as identification of complex structures and matching of objects in an image with specific templates.

#### Digital Image Analysis

Digital image analysis, or just digital imaging, on the other hand, corresponds to the process of extracting meaningful information from an image (2). This information can be descriptive statistics from the image, ranging from global image metrics, such as color/brightness histograms and distribution, block statistics from regions/windows across the images, such as intensity, moments (mean, variance), and integral images, to the identification of more complex structures in the image. Such information extracted from the image analysis can be used then as input for imaging processing techniques, such as image sharpening (4), thresholding (5), smoothing and edge/contour enhancement (6). On the other hand, image processing techniques can also be applied prior to image analysis techniques. An example is the use of edge detection techniques in the process of identification of structures, such as lines and circles in an image (3). Another is for image segmentation, i.e., to divide an image into different regions, which can be simple image binarization (a division of the image into two regions, such as background and foreground) or multiple regions, such as different objects present in an image. There are several methods of image segmentation, but basically, they can be classified into methods that perform a global clustering of image pixels according to some criteria independent of spatial information (e.g., k-means clustering), and methods that account for more information, such as spatial, texture, color, edges, and shape, such as energy-based (graph-cuts) methods (7, 8).

#### Computer Vision

Computer Vision can be defined as the field that aims to describe the world through images by interpreting, reconstructing, and extracting properties from images, such as shapes, textures, densities, and distances (9). CVSs are also known as machine vision systems, visual image systems, or just image systems. Therefore, Computer Vision is essentially the development of artificial systems to handle visual problems of interest, and for such, it uses image processing and analysis techniques. Along with image analysis and processing, other areas such as Machine Learning and Pattern Recognition are also highly interconnected with Computer Vision.

Pattern Recognition is a field that studies not only images but also other signals, such as sound and texts. As the name suggests, it is an area dedicated to the study of patterns that may appear in any given signal. In the context of imaging, pattern recognition is generally studied within image analysis as the development of mathematical methods for the identification of simple geometrical structures such as lines and circles (3, 10) or key-point features that can be jointly used to identify more complex objects or patterns (11, 12). Machine Learning is also a broader field that is concerned with the development and application of algorithms for extracting information from the most diverse data sets (13), and several machine learning algorithms have been developed or adapted specifically for solving computer vision problems.

**Figure 1:**
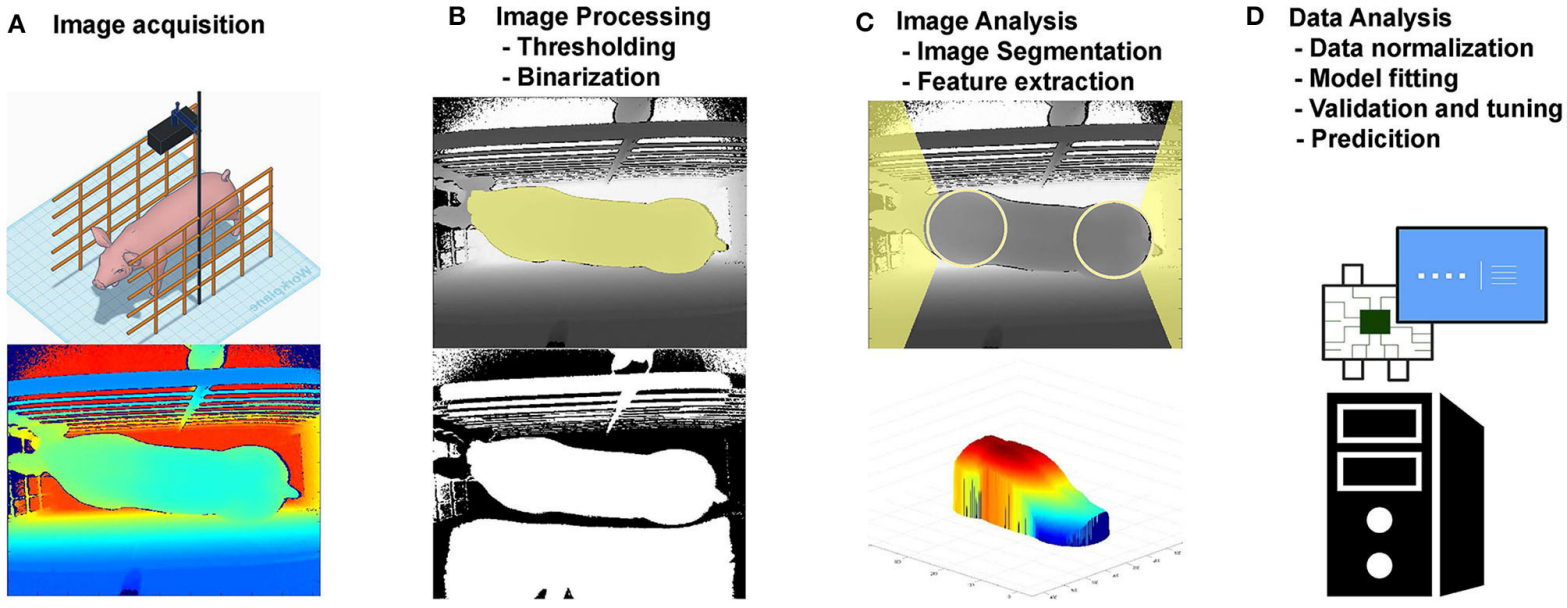
Example of a computer vision system framework. **(A)** Image acquisition; **(B)** Image Processing; **(C)** Image Analysis; **(D)** Data Analysis.

An example of CVS is presented in Figure 1, where a 3D camera is used to capture images from pigs [adapted from (14)]. In a standard pipeline, after these images have been captured, they are processed (Figure 1B) using common imaging processing techniques such as image thresholding and binarization. Using the processed image, features of interest are identified, such as the pig head and tail (Figure 1C), and are removed together with the image background. From the resultant image of the pigs back, several measures were taken (e.g., volume, area, height, and length). These measures leverage important information from the images evaluated and can be used then for the development and evaluation of predictive models (Figure 1D), such as prediction of body weight.

### Image Formation

**Figure 2:**
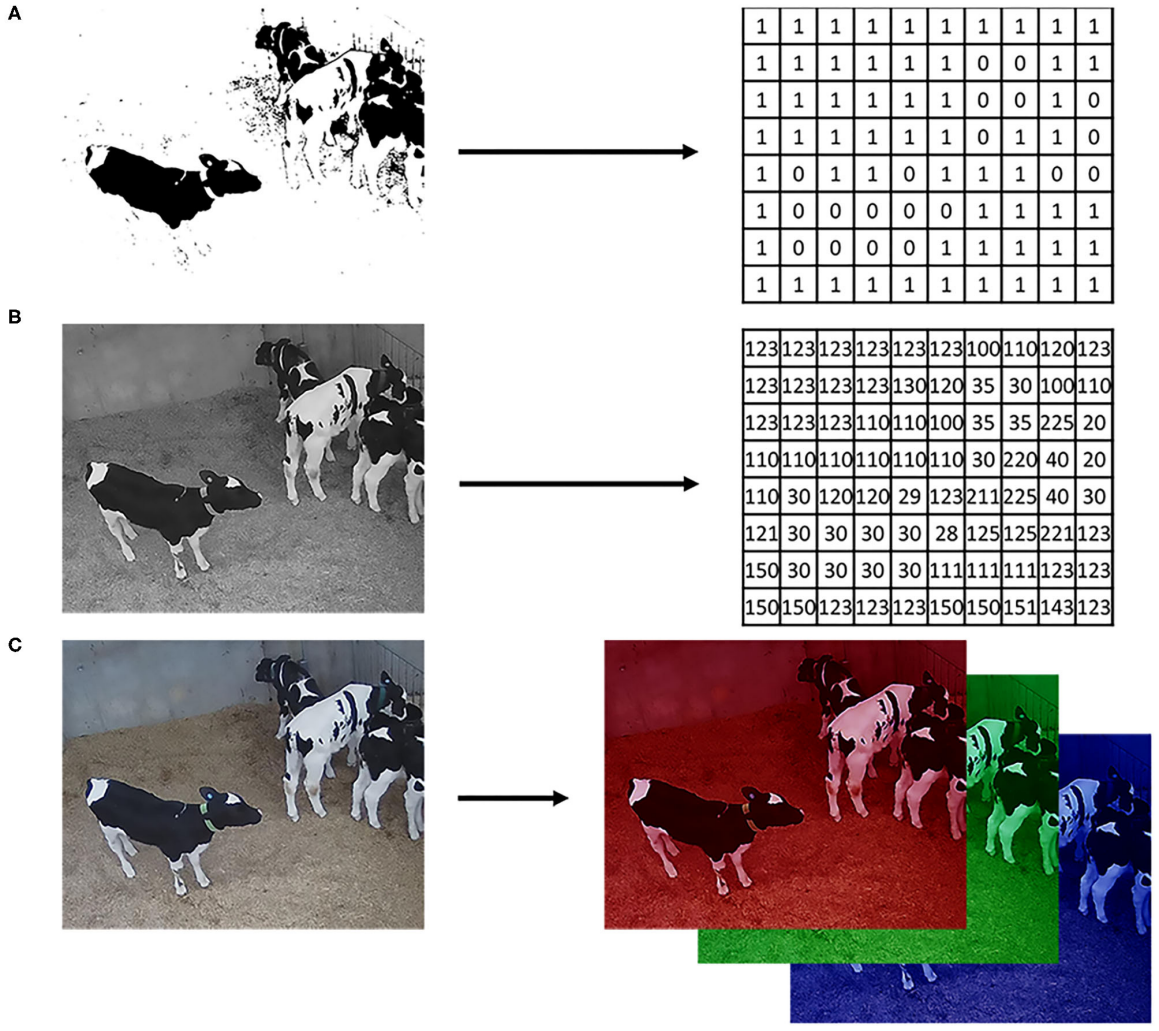
Digital image representation. **(A)** Logical image with values 1 for white and 0 for black; **(B)** Grayscale image in the 24-bits depth format (values ranging from 0 to 255); **(C)** Color image on the RGB color space where each matrix is a 24-bits depth image, one for each color layer.

An important aspect of digital imaging is how the image itself is acquired since there are sensors better suited for different applications. Before images could be processed and analyzed in computers, there was the need to develop sensors able to recognize, measure, and digitalize luminous signals. It was in the 1970s, with the advent of the charge-coupled devices (CCD) sensors (15), that digital imaging was developed, and the interest in CVS appeared. Basically, in digital image formation, luminous signals are captured by the sensor, coded, and stored in arrays of data that can be interpreted and manipulated in computational algorithms (9). Thus, for a computer, an image is nothing more than numerical values in a structured array of data that codifies light and colors for each point in the image (Figure 2). This array can be a single matrix, where the values inside the matrix correspond to black or white (binary image) or different shades of gray (grayscale image). Also, it can be an array of 3 matrices in the case of color images (i.e., intensities of red, green, and blue, on the RGB color space) or even multiple matrices for hyperspectral images. Therefore, mathematical manipulations and statistics of an image were among the first studies developed in digital image analysis and processing.

**Figure 3:**
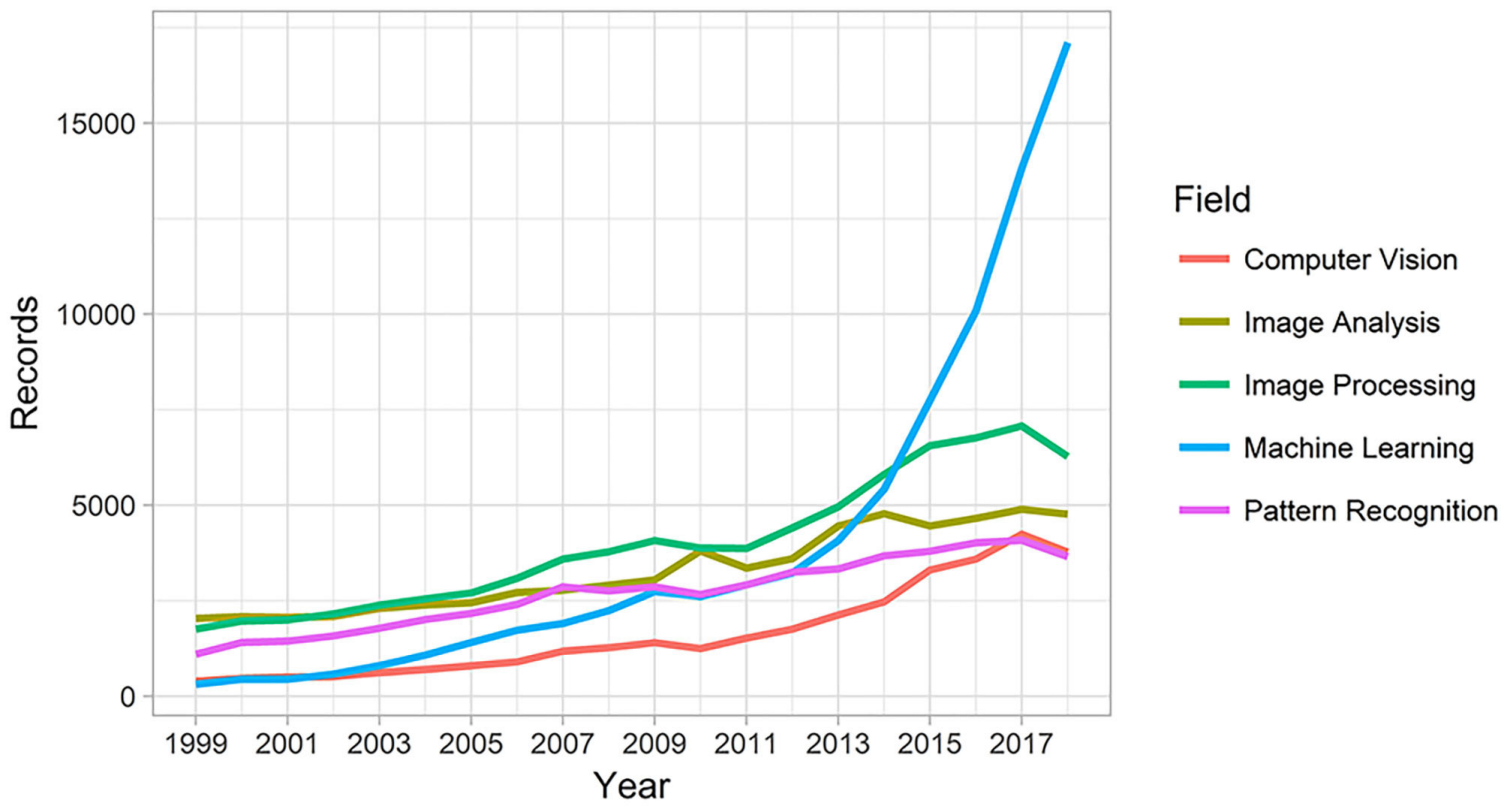
Count of publications hits in “Web of Science” for computer vision, image analysis, image processing, machine learning and pattern recognition.

Another turning point in the history of computer vision was the advent of personal digital cameras in the 1990s, reducing the costs and popularizing the process of capturing and analyzing digital images (16, 17). Since then, several applications of digital photography have appeared. This popularization of digital cameras is directly connected to the increasing volume of data (photos and videos) generated over the last few years in many fields due to the increasing number of computer vision applications to solve the most diverse problems. This increased interest in computer vision and related areas can be illustrated by the increasing number of publications in the last decade (Figure 3).

Some areas of recent interest in computer vision are object sensing, mapping, recognition, motion tracking, navigation, image segmentation, and scene interpretation. However, while humans and animals do most of these actions intuitively, the majority of the vision tasks are considered as difficult problems in computer science, and the algorithms available are prone to errors (9). Thus, many successful CVSs are the result of multidisciplinary approaches tailored for specific cases, for example, interactive segmentation (8), face detection based on image features (18), and machine learning methods for object detection and recognition, such as optical recognition (19) and image classification, such as classification of regions and cells of histopathological images (20).

Among the machine learning techniques used in computer vision, it is worth mentioning deep learning algorithms, which have recently been successfully used in diverse computer vision applications. These algorithms are an extension of traditional artificial neural networks (ANN), and they achieve great power and flexibility by learning more abstract representations of the inputs as a nested hierarchy of concepts (21). These nested concepts, or hidden layers, generate very complex models with many parameters that were possible to be trained only with the advent of very large datasets, data augmentation techniques, and advancements in ANN, such as the development of learning optimization via stochastic gradient descent, new activation functions such as rectified linear unit (ReLU), regularization techniques, and efficient use of graphics processing unit (GPU) (21, 22).

### Metrics for Model Comparison and Assessing Predictive Ability

As there are CVS developed for many different tasks and using a wide range of methods and models, there will be also many ways to compare competing approaches for different applications. In the following, we discuss some of these comparison methods by splitting them according to the class of the variable being predicted. For the scope of this study, we will split the predicted variable into two classes: (1) a variable that we deem associated/correlated to the image, and (2) the image per see (or even parts of the image).

In some applications, the interest will be to predict a variable by using information extracted from the images. These variables of interest can be a categorical variable such as animal species, behavior classes, and scores (e.g., leg score, body conditioning score), or a continuous variable such as body area, height, and weight.

For categorical variables, the simplest case assumes only two states (e.g., health/disease, moving/standing, among others), and the general scenario allows for multiple classes to be evaluated at the same time (e.g., behaviors such as laying, drinking, eating, walking, etc.). The metrics used to evaluate the predictive methods, in this case, are going to assess the frequency of two types of error: false positive (a.k.a. nuisance alarm) and false negative (a.k.a. missing alarm) errors and the most basic assessment tools are via tables of errors, or confusion matrix as below:

**Table:** 

	**y = 0**	**y = 1**
ŷ = 0	TP	FP
ŷ = 1	FN	TN

**Figure 4:**
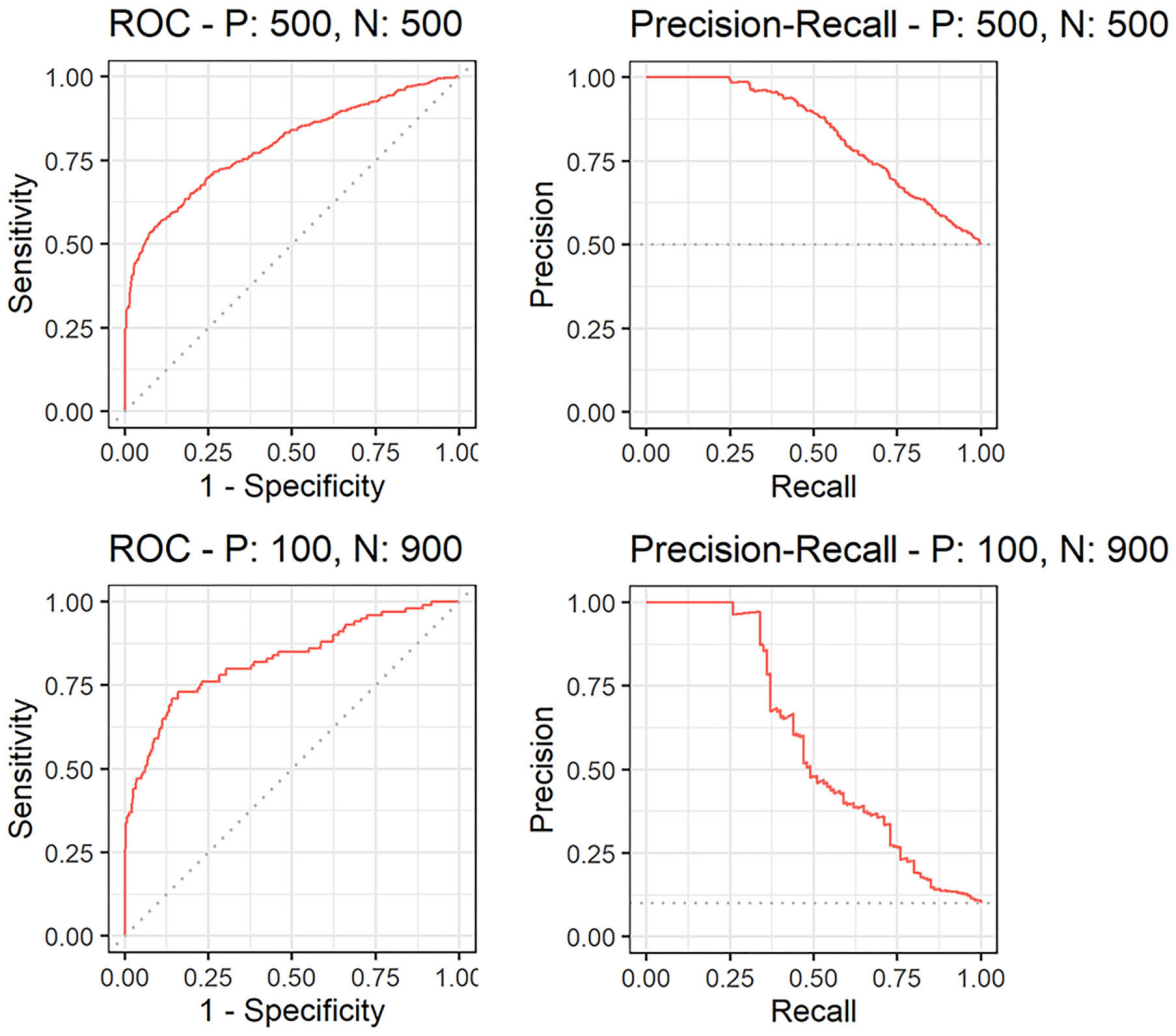
Comparison of receiver operator curves (ROC) and precision-recall curves for a balanced [with 500 positive (P) and 500 negative (N) labels] and unbalanced (with 100 P and 1102 900 N) datasets.

Here, *y* corresponds to the true category or ground truth (e.g., *y* = 1 for disease and *y* = 0 for healthy), which can be a manual measurement or derived from a gold standard method, and ŷ corresponds to the predicted class. The combination of each value of *y* and ŷ gives either a true positive (TP), true negative (TN), false negative (FN), or false positive (FP). From the confusion matrix and the TP, TN, FN, and FP counts for any given experiment, several metrics can be derived, such as: sensitivity (recall or true positive ratio) = $\frac{\text{TP}}{\text{TP}+\text{FN}}$; false positive rate (FPR), also known as, false discovery rate (FDR) = $\frac{\text{FP}}{\text{TP}+\text{FP}}$; precision = $\frac{\text{TP}}{\text{TP}+\text{FP}}$; specificity = $\frac{\text{TN}}{\text{TN}+\text{FP}}$; and accuracy = $\frac{\text{TP}+\text{TN}}{\text{TP}+\text{FP}+\text{TN}+\text{FN}}$. However, when evaluating classification methods sometimes it is interesting to evaluate many threshold values used to classify ŷ as one class or another. This evaluation is often done using receiver operating characteristic (ROC) curves, which measures the tradeoff between sensitivity and FDR, or 1- specificity (which yields the same value). Another metric is the precision-recall (PR) curve which is useful to evaluate the trade-off between precision and recall as the threshold value varies, i.e., the trade-off between the fraction of the detection that is actually true positives (i.e., precision) and the fraction of true positives that are detected (i.e., sensitivity) (13). Also, PR curves are especially interesting when we have situations with unbalanced data. In these situations, a ROC curve may present a misleading high area under the curve (AUC), for a model that is only predicting every data point as from the class that has more true values (Figure 4). For both curves, the quality of competing methods is often summarized by the AUC for which higher area means better fitting, with an area of one meaning a perfect fit.

For applications where the variable of interest is continuous, the predictive ability is typically evaluated using the Pearson product-moment correlation coefficient (r) between the input (*y*) and the predicted output (ŷ):

$$\begin{array}{l} \text{r}==\frac{cov\left(y,ŷ\right)}{\sigma_{y}\sigma_{ŷ}} \end{array}$$

where *cov*(*y*, ŷ) is the covariance, and σ_*y*_ and σ_ŷ_ are the square root of the input and output variances. Alternatively, instead of the predictive correlation r, its square is often reported. Both *r* and r^2^ measure the linear relationship between *y* and ŷ, and the closer to 1 the better. However, they are not measurements of prediction accuracy, as they do not take prediction bias into account. In this context, another measure often reported is the predictive error, or rather, the mean absolute predictive error (MAE or MAPE), which is a direct measure of how much the predictions deviate from the true values and can be defined as:

$$\begin{array}{l} MAE=mean\left(\left|y-ŷ\right|\right) \end{array}$$

Because MAE can be influenced by the scale, when comparing between different studies it is often better to use a scale independent measure (23), such as the mean absolute scaled error (MASE), which can be defined as:

$$\begin{array}{l} MASE=mean\left(\left|\frac{y-ŷ}{mean\left(y\right)}\right|\right) \end{array}$$

In applications where the variable of interest can be the whole images or parts of the image, such as identification of objects within the image, the predictive ability can be evaluated via the pixel-wise accuracy, i.e., the ratio of pixels correctly predicted vs. the total number of pixels. However, this measure of prediction quality will tend to be high for most of the applications as the majority of the pixels within large objects will be correctly predicted. Another interesting measure in this scenario is the Jaccard index, a.k.a. Intersection over Union (IoU), which is the ratio of the intersection between the ground truth (*A*) and the predicted area (Â) by the union of these areas:

$$\begin{array}{l} IoU_{\left(A,\;Â\right)}=\frac{\left|A\cap Â\right|}{\left|A\cup Â\right|} \end{array}$$

Thus, IoU is a measure of similarity between the two areas, and values closer to 1 indicate more similarity, meaning a better fit of the predictive method (24).

As a final note on this topic, it is important to evaluate the generalization performance of the candidate methods, in other words, their predictive capability on independent data set. This evaluation will provide insight into the variability of the predictive error as well as potential overfitting (a situation when a method performs very well on the training data but not so on the independent dataset). This independent dataset, or validation set, is ideally a dataset collected in another moment from different animals. But most of the time if the data is large enough it can be a reserved portion of the original data. Another interesting technique is cross-validation, where the data is divided into multiple subsets, and each time one of the subsets is reserved for validation while the others are used for training. Thus, in a k-fold cross-validation, the dataset is divided into k subsets, and if *k* = *n* (number of data points) the approach is called a leave-one-out cross-validation. For a more in-depth reading on model assessment and selection, the reader can refer to Chapter 7 of Hastie et al. (25).

## Sensors Used for Imaging in Animal and Veterinary Sciences

Currently, the most used image sensor devices are standard digital cameras and/or surveillance cameras that capture electromagnetic waves within the visible light spectra to generate digital images (color or grayscale). However, there are also other technologies that have been used for more specific applications, such as devices that are based on infra-red, ultrasound, and ionizing radiation. Moreover, some technologies can generate more complex arrays of images such as three-dimensional (3D) and hyperspectral images. They are, however, in general, more expensive than standard digital cameras. Nonetheless, each different imaging technology can be used for specific applications.

### Images on the Visible Light Spectrum

Cameras for standard digital imaging work with signals within the visible light range, and they generally have a CCD or a complementary metal-oxide-semiconductor (CMOS) sensor. Both sensors have a similar function, which is to capture light and convert it into a digital image, however, they have some important differences. On one hand, CCD sensors are charged passively by the light source, and the information captured in each pixel is processed sequentially. CMOS sensors, on the other hand, have active pixels with a transistor for each pixel so that the information from each pixel is translated to the image independently and generally asynchronously to the digital image (26). These differences in sensor architecture lead to differences in sensor prices and capabilities. Even though the industry is in constant development, CCD sensors, in general, have a higher dynamic range and produce more uniform images, while CMOS sensors are cheaper, energy efficient, and more responsive.

### Infrared Radiation

Infrared radiation (IR) has a wavelength longer than the visible light, and according to the International Organization for Standardization (ISO) it can be divided into near-infrared (NIR), mid-infrared (MIR), and far-infrared (FIR). This division has been based on the specific wavelength thresholds of 0.78–3, 3–50, and 50–1,000 μm for NIR, MIR, and FIR, respectively. There are many different applications of IR in imaging, and for the purpose of this review, the most significant ones are in 3D imaging, spectroscopy, night vision, and thermal imaging (also known as Thermography). For all these applications, there are different IR sensors specific to capture radiation within NIR, MIR, or FIR ranges. In most of the night vision cameras, the sensors rely on an emitter, which emits IR on the NIR wavelength to actively illuminate the scene. On the other hand, thermal imaging uses the principle that all objects produce radiant heat (emitted, transmitted, and/or reflected), thus there is no need for an emitter since the sensors are capable of capturing the heat signal in the MIR or FIR range (27). The sensors for thermal imaging can be divided into two groups, cooled or uncooled focal plane array. The main difference is that the cooled sensors generally produce better images and less variable measurements, at the cost of being heavier, less portable, and expensive.

In animal, veterinary, and wildlife applications, both night vision and thermal cameras have been used mostly for monitoring animals (livestock or wildlife) at night or dim light situations, either alone or in association with standard digital image sensors (28). Such applications can be dated to the use of military night vision scopes for observation of nighttime animal behavior in the 70s (29). However, thermal cameras also have applications in diagnostic imaging to detect small changes in the body's surface temperature (30), which can be due to stress, fever, inflammation, and ischemia. Nevertheless, proper use of thermal imaging for diagnostic purposes still requires correct calibration of the device, adequate location, and correct positioning of the animal and camera (27, 30).

### 3D Imaging

Many different sensors and techniques are used for measurement of the distance of objects to the camera, acquisition, and formation of 3D images. In livestock, these sensors can be used, for example, for measurement of animal volume, surface, and gait, among other traits. From the several technologies developed for 3D imaging, we will focus on optical applications (i.e., applications that use radiation on visible and near-visible light) used in 3D cameras, also known as depth sensors. These techniques can be further divided into passive, such as *stereo imaging* and *structure from motion*, or active, such as *structured light* and *time of flight* (31).

**Figure 5:**
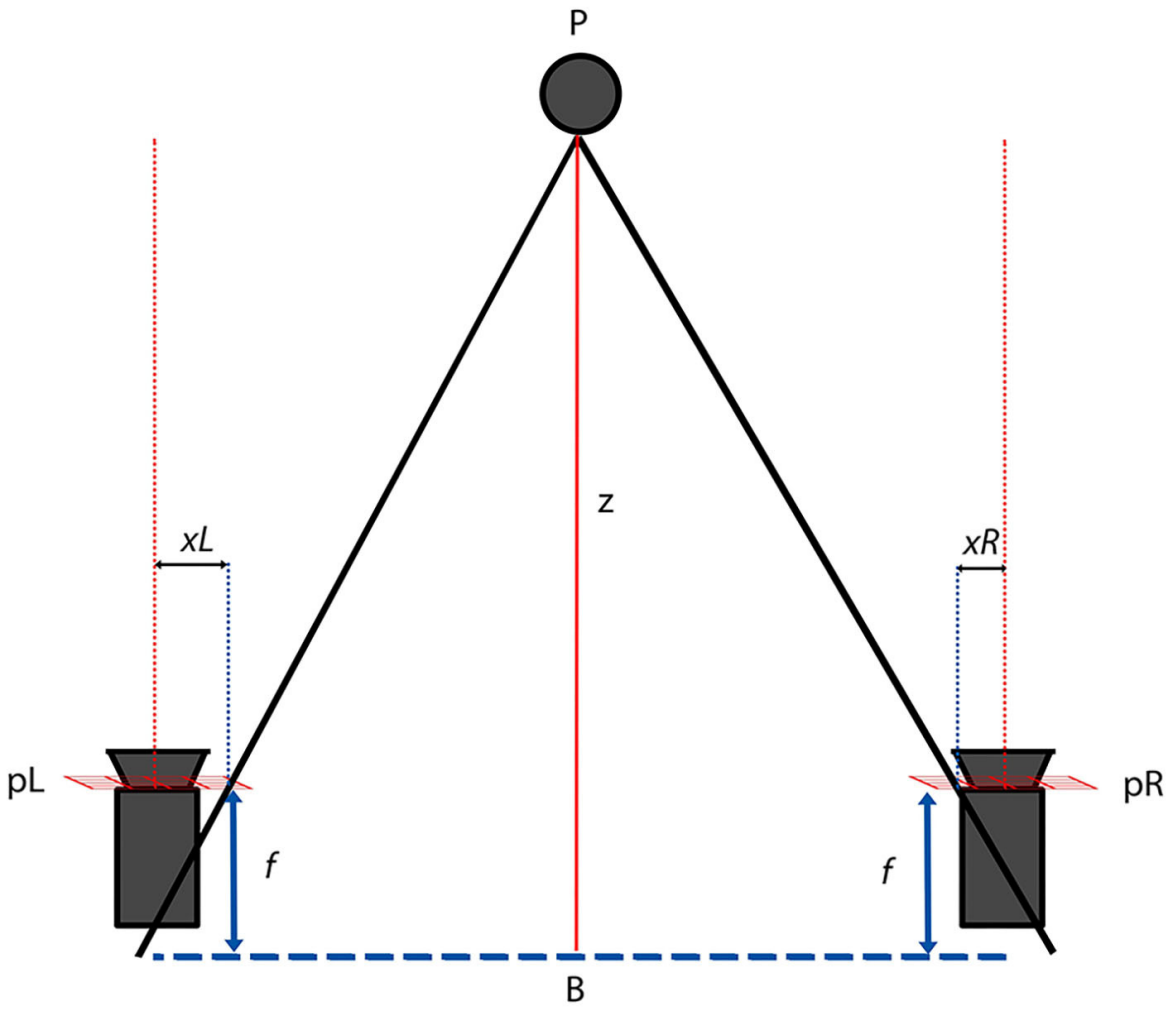
Epipolar triangulation used on a rectified stereo imaging system with two similar cameras. P is the point of interest; xL and xR its projection on the camera planes pL and pR; f is the camera focal length and B the baseline plane.

In *stereo imaging*, two or more cameras are used, and principles of epipolar geometry are applied in order to calculate the distance (*z*) of a point P to the cameras (32). In a simplified stereoscopic triangulation (Figure 5) using two similar cameras with the same focal length *f*, the differences of the projections (*x*_*L*_and *x*_*R*_) of point P on the planes of the cameras is the disparity between the images. That disparity can be used then to calculate the distance of point P to the baseline plane, where B is the distance between cameras.

Similarly, in *structure from motion*, a disparity map can be created between the images from a single moving camera, in which case the distance between the points where each image was captured by the camera can be used as the distance between “cameras.” The main difficulty of such a strategy is that it needs the object of interest to be practically motionless.

**Figure 6:**
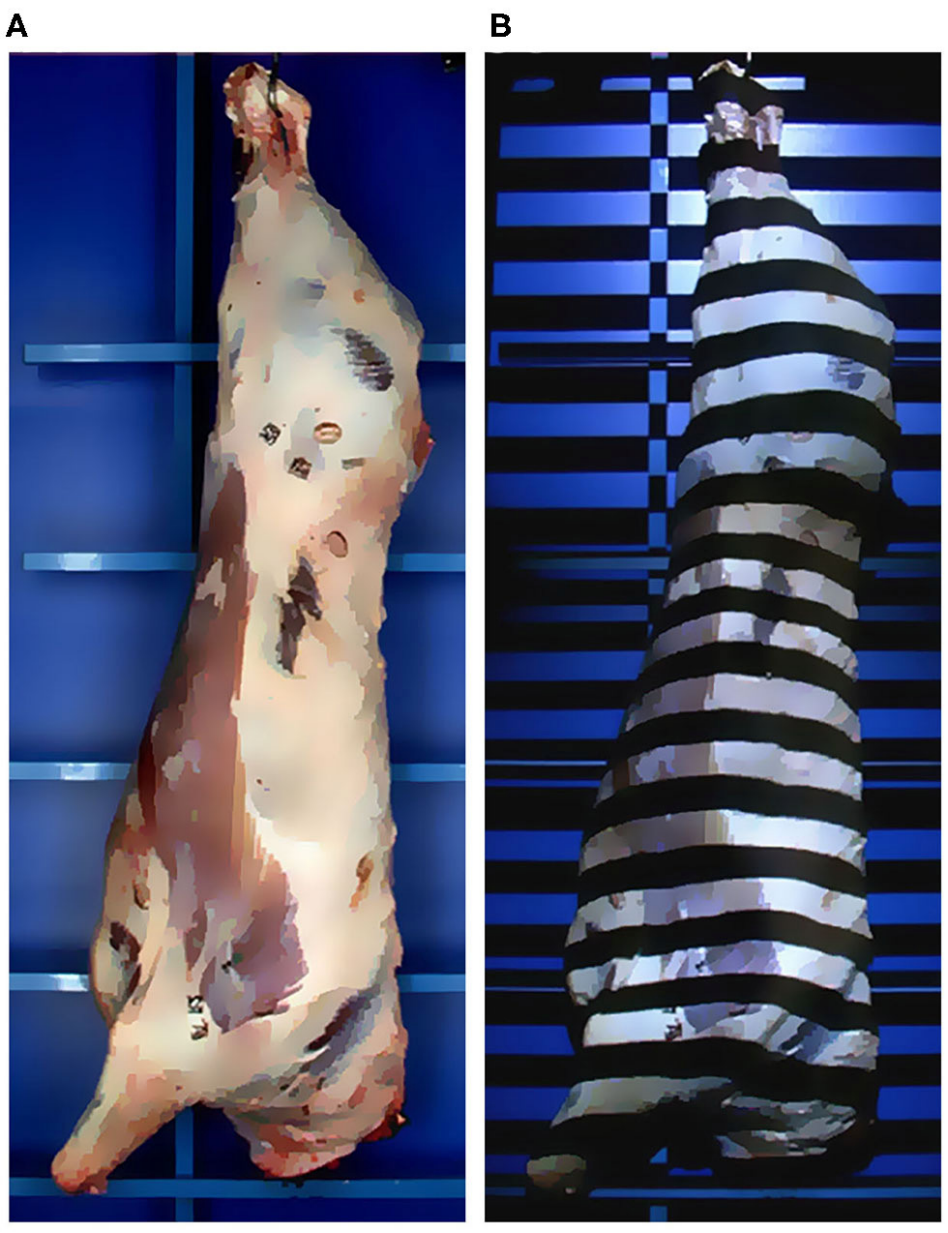
Example of a structured light system based on linear shadow pattern. **(A)** The scene with natural illumination. **(B)** The same scene now under the structured light projected by the emitter.

*Structured light*, also known as *coded light* refers to the use of active emission of known light patterns for which the illuminated surface will present structural distortions in the shade/light patterns according to irregularities in the surface, angle, and distance to the emitter (Figure 6). Therefore, similarly to *stereo imaging*, the distortions in the emitted patterns provide unique correspondence for triangulation with the camera. The emitter can vary from a punctual laser, a blade scanner, multiple shadow patterns that split the scene into areas of interest, or even the use of complex multi-laser patterns that create a spatial neighborhood (31, 32).

**Figure 7:**
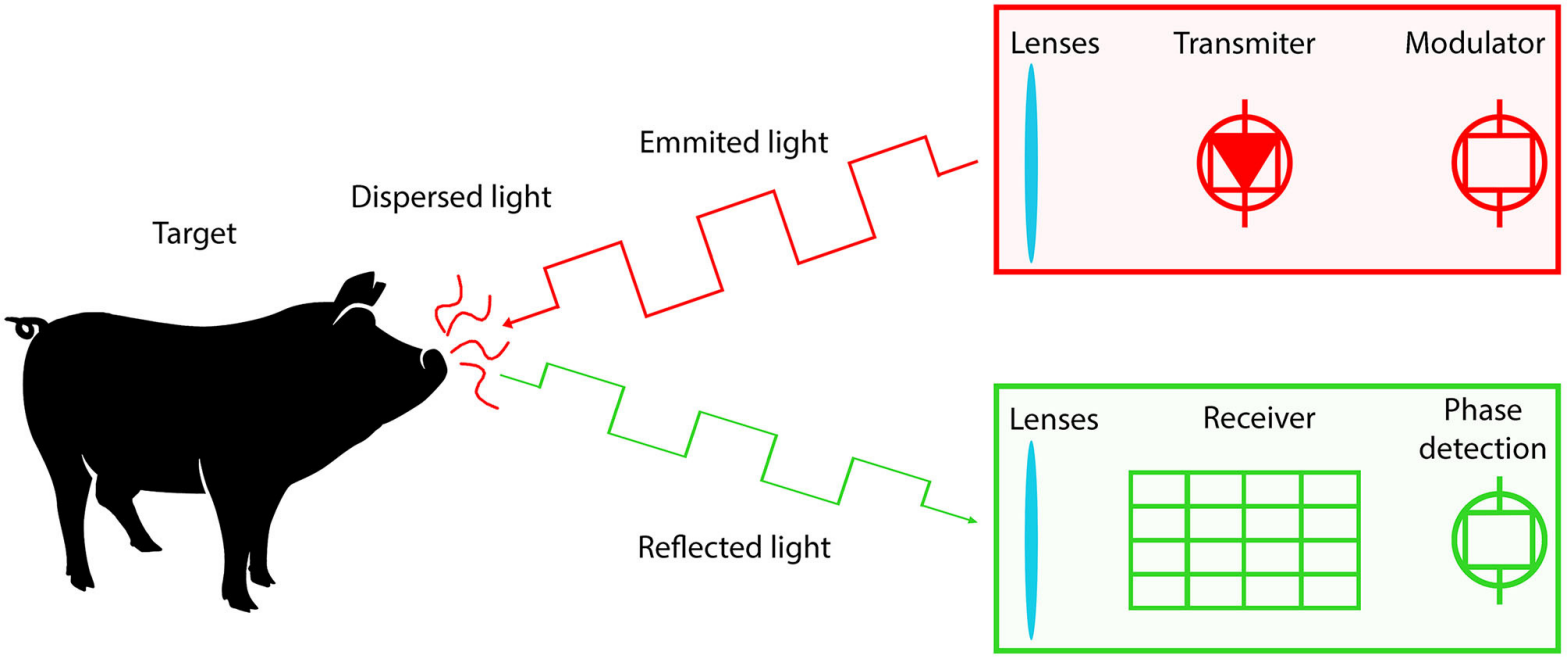
Principle of time of flight (ToF) 3D cameras (depth sensors).

*Time of flight* (ToF) and *Light Detection and Ranging* (LiDAR) cameras are based on signal modulation and ranging, similar to other technologies such as *Sound Navigation Ranging* (SONAR) and *Radio Detection and Ranging* (RADAR) (32). These techniques measure the distance between the sensor and a target object by detecting the time difference from the signal emitted by a transmitter, reflected on a target object, and captured back by a receiver (Figure 7). Even though the principle is simple, there is a great implementation challenge due to the speed of light, interference with natural light, dispersion, and absorption of the light. Modern ToF cameras generally consist of a transmitter array that emits a modulated IR or NIR light (to reduce environmental interference) and a receiver array that captures the reflected signal and calculates the signal phase/time lag for each pixel (31). Recently, in order to improve the transmitter performance, devices equipped with micro-electro-mechanical system (MEMS) mirrors have received great interest from the scientific and industry community (33).

Lastly, there are also hybrid 3D cameras that combine RGB sensors with depth sensors based on one or more of the technologies described above. Examples include cameras based on active stereoscopy, which combine stereo imaging from multiple cameras with structured light to improve the depth estimation. Table 1 shows some of the current 3D cameras available and their technical specifications.

**Table 1:** Comparison of 3D cameras and their technical specifications.

**Device**	**Manufacturer**	**Sensors**	**Technology**	**Range (m)**	**Environment**	**FPS**	**FOV (V × H)**	**Resolution (pixels)**
Kinect V1^a^	Microsoft	3D (IR emitter + IR camera) Color	Structured Light	0.8– 4	Indoor	30 30	45° × 58°	480 × 640
Kinect V2^a^	Microsoft	3D (IR emitter + IR camera) Color	Time of flight	0.5–4.5	Indoor	30 30	60° × 70° 54° × 84°	424 × 515 1080 × 1920
Kinect Azure	Microsoft	3D-N (IR emitter + IR camera) 3D-W (IR emitter + IR camera) Color	Time of flight Time of flight	0.5–5.5 0.3–2.8	Indoor	30 30	65° × 75° 120° × 120° 59° × 90°	576 × 640 1024 × 1024 2160 × 3840
Xtion^a^	Asus	3D	Time of flight	0.8–3.5	Indoor	30	45° × 58°	480 × 640
Xtion Pro Live	Asus	3D Color	Time of flight	0.8–3.5	Indoor	30 30	45° × 58°	480 × 640 1024 × 1280
Xtion 2	Asus	3D Color	Time of flight	0.8–3.5	Indoor	30 15	52° × 74° 60° × 75°	480 × 640 1944 × 2592
Intel SR305	Intel	3D (IR emitter + IR camera) Color	Structured light	0.2–1.5	Indoor	60 30	54° × 70° 42° × 68°	480 × 640 1080 × 1920
Intel D415	Intel	3D (IR emitter + IR camera) Color	Active Stereo	0.2–10	indoor/outdoor	90 30	40° × 65° 43° × 70°	720 × 1280 1080 × 1920
Intel D435	Intel	3D (IR emitter + IR camera) Color	Active Stereo	0.1–10	indoor/outdoor	90 30	58° × 87° 43° × 70°	720 × 1280 1080 × 1920
Intel L515	Intel	3D (IR emitter + MEMS^b^) Color	LIDAR	0.2–9.0	Indoor	30 30	55° × 70° 43° × 70°	768 × 1024 1080 × 1920
Structure^a^	Occipital	3D (IR emitter + IR camera)	Structured Light	0.8–4.0	Indoor	30	43° × 57°	480 × 640
Structure II	Occipital	3D (IR emitter + IR camera) Ultra-wide monochrome	Active Stereo	0.3–5.0	indoor/outdoor	54 100	46° × 59° 160°Diag	960 × 1280 480 × 640
Structure Core	Occipital	3D (IR emitter + IR camera) Color	Active Stereo	0.3–10	indoor/outdoor	54 100	46° × 59° 85°Diag	960 × 1280 480 × 640

a *Out of production/Discontinued; ^b^Micro-electro-mechanical system mirror*.

### Other Imaging Technologies of Importance

There are many other imaging related technologies of importance in animal and veterinary sciences, such as spectral and hyperspectral imaging, ultrasound, x-ray, computed tomography, and satellite images. In the following, we briefly describe some of these technologies.

Spectroscopy, spectral imaging, and more recently hyperspectral imaging have been adopted extensively for evaluation of meat attributes and chemical characteristics as well for quantification of milk protein and fat content (34, 35). These technologies are mostly composed of sensors equipped with a NIR emitter, and are based on the principle that different compounds will absorb the radiation differently in each wavelength, thus generating a “signature” (36). In Spectroscopy, for each wavelength measured inside the range, a punctual value of absorbance is generated. Meanwhile, a spectral image is a matrix with a value of absorbance for each pixel, and a hyperspectral image corresponds to a cube of several matrices (one for each wavelength) providing both spectral and spatial information.

Several medical diagnostic imaging technologies have also been employed for many applications with animals such as evaluation of muscle and fat composition, and bone mineralization in live animals and carcasses (37–39). Some worth noting technologies include ultrasound (US), dual-energy x-ray absorptiometry (DXA), computed tomography (CT), and magnetic resonance (MR). All these technologies are appealing since they make possible the generation of images for the evaluation of the body composition. From these technologies, only US is currently used in farm conditions due to many factors such as price, portability, and no anesthesia required. Nonetheless, it still requires a trained operator.

## Applications of Computer Vision Systems in Animal and Veterinary Sciences

Before the advent of CVS, many applications required the use of the trained eye for visual classification of traits in live animals, such as animal behavior, body condition score, carcass fat deposition, meat marbling, or classification of eggshell quality. There are also methods that use the aid of lenses, such as microscopes, for evaluation of cell morphology in a blood smear or spermatozoid motility and defects. Moreover, other signals such as ultrasound, infrared, and x-rays are widely used to produce images for diagnostic purposes. However, most of the methods currently used for the measurement of traits of interest need expert personnel requiring the training of evaluators from time to time to maintain good measurement quality. Also, most of such measuring processes are time demanding, stressful to the animals, and costly for the farmer, making it prohibitive due to animal welfare and economic reasons. Therefore, there is an interest in developing automatic, indirect methods for monitoring livestock and measuring traits of interest. For such tasks, CVS generally uses algorithms and principles of pattern recognition, image analysis, and processing in order to tackle the most diverse problems. The framework presented in Figure 1 can be seen as a CVS pipeline, with a fixed sensor capturing the information that is presented by the world or actively exploring the world and adjusting its perception (field of view, exposure, among others). The development of automated CVS can enable high-throughput phenotyping in livestock, and the data generated by such systems can be then used for many different applications, from the development of smart farm management tools to advancing breeding programs.

In the following, we present selected applications of CVS as an answer to the need for such automated, non-invasive methods for the measurement of carcass and meat traits, live animals' identification, tracking, monitoring, and phenotyping using different sensors.

### Carcass and Meat Traits

Probably one of the first applications of a CVS was in meat sciences, with the earliest reported studies found in the 1980s (40–42). In these studies, the system was composed of a camera, light source, digitizer, and computer unit. The CVS needed an operator to position beef meat cuts on a surface at a known distance and angle from the camera, and to trigger the image acquisition. Thus, the meat cuts were all positioned in the same manner with constant background and illumination. At that time, the interest was to predict the cut content of lean meat and fat, and to compare the results from the CVS to trained United States Department of Agriculture (USDA) meat graders. In these studies, multiple linear regressions fitting variables extracted from the CVS were compared to models that included manual measurements and USDA graded variables. Even though the system was not fully automated, the prediction equations developed with the variables measured by the system presented slightly better results than the prediction equations developed with variables measured by trained graders. The best linear model for prediction of lean meat weight developed with the variables extracted from the CVS achieved *R*^2^ from 0.93 to 0.95 against 0.84–0.94 from the model that included USDA graded covariates (41, 42).

Since these earlier studies, there has been an increasing interest in the use of computer vision for prediction of the most diverse meat quality traits, not only for beef but also for fish, poultry, and pork (Table 2). There are applications focused on imaging technologies for determination of not only meat crude protein and fat content but also more refined chemical characteristics like fatty acids profile, freshness (50, 57), as well as prediction of meat quality, palatability, tenderness, and other traits normally evaluated by a panel of trained experts (45, 46, 51) or even automatic sorting and weighing cuts and viscera which is normally performed manually (48, 52). Again, different devices and imaging technologies have been used, with several predictive approaches evaluated. However, independently of the imaging device used, such applications pipelines generally involve several steps such as: (1) Sample preparation with standardization of meat cut used, presentation, background and light conditions; (2) Device calibration (when needed), collection and processing of the images; (3) Direct measurement of attributes of interest using a gold standard methodology (i.e., chemical analysis); and (4) Model fitting, which corresponds to the prediction of the gold standard using the image features as predictors. It is interesting to note that the image processing in step 2 can involve several sub-steps such as histogram equalization, background removal, and image smoothing. Also, in the case of hyperspectral images the processing involves selection of wavelengths and/or reduction of dimensionality using techniques such as Fourier transformation (58) and principal components, for an in-depth review of applications of hyperspectral imaging see Xiong et al. (34). It is also worth noting, that there is no standard model of choice for step 4 since in the literature several predictive approaches have been evaluated. These predictive approaches could be statistical models such as linear and partial least square regression to machine learning methods, such as support vector machines, random forests, and artificial neural networks.

**Table 2:** Examples of computer vision applications in meat sciences (studies highlighted in bold were with live animals).

	**Applications**	**Image signal**	**References**
Cattle and Small Ruminants	Carcass	3D; US; VL	(41, 43, 44)
	Fat (kg and%)	US, VL	(41, 43, 45)
	Lean meat (kg and %)	VL	(41, 42, 45)
	Tenderness	VL	(45, 46)
Fishery	Fat Pigmentation	IR; VL	(47)
	Sorting	HS	(48)
	Freshness	VL; HS; 3D	(49, 50)
Poultry	Classification	HS; VL	(51)
	Brest weight	3D	(52)
	Egg shell classification	VL	(53)
Pork	Carcass	US; VL; CT; 3D	(37, 39, 54–56)
	Classification	HS; VL	(51)
	Quality	HS; IR; VL	(57)

*CT, Computed Tomography; 3D, 3-dimensional; HS, Hyperspectral; IR, Infrared; US, Ultrasound; VL, Visible Light*.

### Monitoring and Phenotyping of Live Animals

Differently from carcass and meat cuts that can be easily positioned for image acquisition under a well-controlled light source and even background, several obstacles arise when working with live animals. As an example, in farm conditions, the illumination can change throughout the day even inside a barn due to sun position, clouds, and seasons. Moreover, there will be differences between artificial light sources from one farm/barn to another, as they may use different types of lamps with different voltage and positioning. The background is also going to be different in each location, and it is prone to changes over time for a given location. Examples of differences in the background are floor surface material for animals in a barn and vegetation for free-range animals. Therefore, the diversity of situations is probably one of the biggest challenges in implementing a CVS that are robust enough to perform satisfactorily across different farm conditions.

Nevertheless, many efforts have been made over the years to develop CVS for monitoring and phenotyping livestock, poultry, and fish as well. In the current study, we do not intend to deliver an extensive review on the matter as there are already reviews on technology applications for poultry (59, 60), machine vision for detection of cattle and pig behavior (61), and computer vision applications for fisheries (62). Nonetheless, in the following, a broader view is presented regarding applications developed for traits of interest in animal and veterinary sciences, providing examples from earlier works to the current trends while highlighting challenges, advances that have been made, and areas of current interest. Table 3 shows a summary of selected applications, presenting the traits of interest and the kind of imaging sensor used.

**Table 3:** Examples of computer vision applications in live animals.

	**Applications**	**Image signal**	**References**
Cattle and small ruminants	Mastitis	IR	(63–66)
	Digital dermatitis	IR	(67, 68)
	Body temperature	TR	(69–71)
	Gait and body measurements	3D	(44, 72)
	Weight	3D	(44, 73)
	Coat and conformation	VL	(74)
	Body condition	VL; TR; 3D	(75–78)
Fishery	Tracking	3D	(79)
	Shape	VL	(80)
	Weight	VL	(81)
Poultry	Behavior	VL; 3D	(82–84)
	Shape	3D	(84)
Dog	Behavior	3D	(85)
Pork	Tracking	VL; 3D	(86–90)
	Behavior	VL; 3D	(91–94)
	Weight	VL; 3D	(14, 95–97)
	Gait and body measurements	3D	(14, 97–99)

*3D, 3-dimensional; TR, Thermography; VL, Visible Light*.

#### Evaluation of Body Composition, Meat, and Carcass Traits in Live Animals

In section Carcass and Meat Traits we saw that CVS had been successfully used for prediction of traits such as lean meat and fat content from carcass and meat cuts. Nevertheless, the same predictive performance has not been observed initially for the evaluation of meat and carcass traits in live animals.

Initial attempts for the prediction of carcass composition on swine have been performed by Doeschl-Wilson et al. (54) where a CVS achieved a predictive *R*^2^ of 0.31 and 0.19 for fat and of 0.04 and 0.18 for lean meat on the foreloin and hindloin regions, respectively. In the search for improvement of performance on the prediction of carcass and meat traits on live animals, researchers have focused on the use of medical imaging devices for such tasks. In recent studies using medical imaging devices, ultrasound measurements presented positive and moderate correlations of 0.6 and 0.56 with the carcass measurements of lean meat and fat depths, while CT presented low to moderate correlations of 0.48–0.67 for fat and high correlations of 0.91–0.94 for lean meat (37, 39). Moreover, these technologies have several drawbacks regarding animal handling and cost, as explained previously. In order to tackle these limitations, some recent works developed CVS based on 3D cameras for non-contact automated estimation of muscle score (55) and of fat and lean muscle content (56) on live pigs. Alsahaf et al. (55) developed a system that extracted morphometric features from the images of moving pigs for prediction of muscle scores between 1 and 5. With a gradient boosted classifier, they achieved classification accuracy between 0.3 and 0.58, and MAE of 0.65, showing that most of the errors where between neighboring classes. Meanwhile, Fernandes et al. (56) evaluated not only features extracted from the images, but also deep learning methods that do not require image processing, with the deep learning approaches achieving better results. Their results present an improvement over previous studies with cross-validation predictive MAE, and *R*^2^ for lean muscle depth of 3.33 mm and 0.50, respectively, and of 0.84 mm and 0.45 for fat depth. Nevertheless, these *R*^2^ presented are still low showing and there is room for improvement on the predictions of lean muscle and fat.

#### Animal Tracking and Behavior Analysis Using CCD or CMOS Cameras

Some of the most desired applications of CVS for live animals correspond to animal identification, tracking, and monitoring, ultimately identifying changes in their daily behavior. Animal identification can refer to the identification of an animal when there is only one animal in the image to more complex scenarios where there are multiple animals in the image or the identification of different individual animals in a single or multiple images matching their identification. Meanwhile, tracking involves the continuous identification of the animal across frames in a video feed or across images from different locations as the animals are moved from one location to the other. Regarding behavior, animals tend to synchronize their behavior within a group, and conspicuous deviations may be caused by environmental stress, management problems, or disease, although individual behavioral differences need to be taken into account. Therefore, there is a constant effort to understand behavioral changes and their relationship with other traits of interests, such as animal health status and growth. Closer evaluations of animal behavior and health are normally conducted by trained evaluators at specific time points, such as the time of transferring animals from one location to another (e.g., from nursery to grow-out farms) or around vaccinations. This is because managers and workers have limited time to spend in observing a group of animals. Also, with the current trend of an increasing size of livestock operations, there is also an increase in the animal/manager ratio. Thus, a basic use of CVS for evaluation of animal behavior can be the acquisition and storage of images and videos that can be assessed later or remotely by the farmers. This improves animal management since there is no need for the evaluator to be physically present, which otherwise can cause behavioral changes on the animals. Also, the evaluator can loop across images, and replay them, improving the quality of the evaluation. Nonetheless, this kind of system is not optimum since the evaluator would still need to check all the images. Therefore, there are efforts in the literature that attempt to develop CVS that can automatically classify animal behavior and alert the manager in real time regarding important changes.

Initial works with pigs demonstrated the applicability of a CVS to identify the animal position and to track its movement (86, 87). These works showed that the image processing algorithms available at the time could segment a single pig from the background under specific conditions. The conditions were: (1) camera positioned to get the top view of the animal, and (2) dark background for a white pig. The method developed by Tillett et al. (86) estimated a point distribution of landmarks on the pig contour for a sequence of frames (13–30 frames) and was able to model small changes in the animal's posture. However, only seven sequences where evaluated and it was prone to miss the animal if the changes in position were abrupt from one frame to another. On the other hand, Lind et al. (87) used a more robust segmentation approach based on the generation of a background matrix for image subtraction and consequently animal segmentation. Even though this method cannot identify animal posture, it was efficient for use in a real-time application and efficiently tracked differences in animal activity. In their study, the developed CVS was able to track the distance traveled and the walking behavior (path) identifying differences between a pig that received apomorphine or not. Similar approaches based on traditional imaging thresholding and frame by frame comparisons were also used with broilers for the identification of flock behavior over time (82) and at different feeders (83).

Animal tracking and activity-related traits are still of great interest, with the identification and tracking of multiple animals and their interactions as one of the biggest issues. In order to overcome this challenge, a successful approach in pigs was to identify the animals by ellipsis fitting on the animal area and recognition of patterns printed on their backs (88). By using this simple approach, researchers were able to track and identify multiple animals with an accuracy of 88.7%, enabling the characterization and evaluation of simple activity status as active or non-active with high correlation (mean of 0.9) with evaluations made by a human observer (88, 100). However, this identification and tracking approach cannot be used for animals with darker skin or in commercial farms that have animals with different skin colors since the method was developed for white pigs on a dark floor background and using a surveillance camera. Another challenge in the use of patterns printed on the animal's skin for identification in commercial settings is the higher stocking density and pen size. In an attempt to solve the issue of multiple animal tracking, Matthews et al. (89) developed an approach using multiple 3D cameras to track multiple pigs in a pen and record their behavior, achieving an overall tracking accuracy of 0.89. In a more recent study, a deep learning approach has been tested for identification and tracking of multiple pigs using standard digital cameras, achieving a precision of 0.91 and recall rate of 0.67 on a test data set of pigs under challenging floor and lightening conditions (90). Even though these are promising results, these systems are prone to lose animal tracking over time, without a current solution on how to get back to the correct tracking of each animal. Thus, there is still the need for a reliable CVS capable of identification and tracking of individual animals in farm conditions.

In order to identify more behaviors, like feeding and drinking, manual segmentation of the captured image in regions of interest (ROI) have proved effective (91). The basic concept is to identify not only the animals but also objects, such as the water source and feeders, and track how animals interact with those objects. Using this technique associated with a transfer function model, with a single input and single output, Kashiha et al. (91) were able to identify pig drinking behavior with an *R*^2^ of 0.92 on a single dataset with 40 pigs divided in 4 pens. Machine learning techniques have proven efficient for the identification of animal posture such as standing, lying, or sitting (85, 93). In their study, Barnard et al. (85) achieved a mean accuracy of 0.91 when using a structural support vector machine to classify dog postures from depth images. In another study, Lao et al. (93) defined a classification tree for identification of several sow behaviors using videos from 3D cameras with high (99%) accuracy for lying, sitting, and drinking behaviors and lower for kneeling (78%) and shifting (64%). Machine learning techniques have also shown to be powerful for the identification of social interactions among animals, such as mounting and aggressive behavior (92, 94). Viazzi et al. (92) achieved a mean accuracy of 0.88 when using linear discriminant analysis for classifying aggressive behavior in pigs, while Chen et al. (94) achieved an accuracy of 0.97 on the validation set using a convolution neural network and long short-term memory approach. Even though there was an improvement in accuracy in the latter study, it did not include an automated strategy for the identification of individual animals. Thus, current methods can be used for the identification of behavior changes on group level, but not on individual level. Another aspect that must be highlighted here is that the methods discussed above are supervised learning approaches, and as such, they need a dataset of labeled images (ground truth) for the training step. In order to produce those training datasets, manual classification of the images by a human observer is needed, thus the model will be at most as good as the human observer who evaluated the images in the first place. One way to improve the gold standard used in such methods is by the development and adoption of well-defined methodologies for the measurement and record of traits of interest, followed by regular training and testing of the human evaluators to increase intra and inter-evaluator reliability. Another approach that can also be used is crowdsourcing the development of the dataset. With crowdsourcing, the manual classification of the images can be done by several evaluators and using majority vote, so reducing the impact of individual evaluators' subjectivity (101). Another approach that has shown improvement of the predictive accuracy is the use of multi-model prediction, also known as model assemble methods. One of the most basic assemble would be the use of the average predicted value from multiple models (102). The combination of crowdsourcing the dataset development with the use of multiple model classifiers has allowed an increase in accuracy for applications in medical image analysis, with an artificial intelligence system outperforming trained evaluators (103).

#### Identification of Mastitis and Digital Dermatitis by Thermography

So far, most of the computer vision applications presented have used standard CCD or CMOS cameras. As previously discussed in section Images on the Visible Light Spectrum, there are also other sensors of interest, such as thermal and depth cameras. Thermal imaging cameras are commonly used in veterinary sciences as a diagnostic tool in clinical examination. The images can be used to identify differences in external/skin temperature that can be related to inflammatory process, infection, necrosis, stress, and overall health. In research, infrared thermography (IRT) has been used to identify mastitis in dairy cattle and sheep (63, 64), and for digital dermatitis in sheep (68), showing the capability to classify healthy and clinically sick animals. Moreover, in a controlled study Metzner et al. (65) observed that IRT was capable of detecting an increase in udder temperature ~10 h after inoculation with *E. coli*. Nevertheless, these were clinical trial studies with a limited number of animals, thus there is still the need to evaluate IRT under more general farm conditions. Zaninelli et al. (66) evaluated the use of IRT for udder health using data from more than 300 cows from three farms. In their study, even though the images were collected manually, the imaging processing was automated using a classical image threshold for measurement of udder temperature. In this initial step toward automation of udder health evaluation, a threshold model was developed for the classification of udder health in two categories, achieving an area under the curve (AUC) around 0.8. In another study, 149 cows from eight farms were clinically evaluated for digital dermatitis, and IRT was evaluated as a non-invasive field diagnostic tool for dairy cattle (67). In their study, an AUC of 0.84 for the receiver operating characteristic (ROC) curve was observed for classification on the temperature difference from the front and rear feet, showing promises of IRT as an on-farm tool. However, the authors pointed out that images were collected manually and that in 11% of the cows' data had to be removed due to excessive dirt. Also, there are still many complications related to IRT measurement variation and repeatability. In some studies, it was observed that animal skin/surface temperature can vary according to external factors, such as environmental temperature, wind speed, or other factors, such as operator and camera positioning, and body region evaluated (69–71). Thus, IRT applications have been challenging, and they are still generally based on semi-automated CVS, so that additional effort should be placed on developing methods for automation and improvement of measurements for on farm conditions.

#### Evaluation of Animal Surface and Related Traits Using 3D Cameras

The interest in the use of 3D cameras is due to the capability of measuring traits in the 3-dimensional space such as animal, body position, gait, and volume. Also, for some applications, there is an improvement of image processing since, within 3D images, there is less noise due to light and background conditions and it is easier to use the distance to the camera as a threshold. Regarding the uses of depth sensors, there are many examples of successful applications including tracking of fish within a tank (79), identification of landmarks on animal shape with consecutive modeling of gait (72, 99), body condition score (78), sickness detection (84), and the estimation of many other body measurements that will be discussed below.

Studies in gait analysis usually demanded intense manual labeling of video frames by a human observer and/or an expensive system of plate markers to be positioned on the animal body and multiple cameras (98). However, with the introduction of time of flight technology and more accessible 3D cameras, CVS with a single or two sensors were capable of efficiently estimate walking kinematics in pigs in a cost-effective framework with prediction accuracy comparable to the state of the art of kinematics systems (*R*^2^ = 0.99) (99).

Spoliansky et al. (78) developed an automated CVS based on 3D cameras for the evaluation of dairy cows' body condition score (BCS). In this study, top view images were collected from cows at the moment they were leaving the milking parlor. These images were then automatically processed with removal of background, rotation, and centralization of the cow, holes filled, and normalization. Several image features were extracted from the processed images and used for the development of multiple linear regression models via stepwise regression. Even though the variables extracted did not present a high correlation with BCS, the developed model achieved an average *R*^2^ of 0.68, which is comparable or better than previous studies based on manual processing of the images using either standard digital images (75, 76) or thermal cameras (77).

In chickens, 3D sensors have also been used to identify small modifications in the animal surface that is related to head and tail positioning (84). In this study, in which animals were challenged with the Newcastle disease virus, it was possible to identify alterations in the animal shape and behavior 6 days after the inoculation.

Other applications in which depth sensors are showing promising results are for estimation of animal body measurements (heights, widths, area, and volume as examples) and body weight (44, 96, 97). In one study, a CVS based on depth image could extract additional information on the animal volume, achieving an *R*^2^ of 0.99 (96) under experimental conditions. In another study (97), depth cameras were evaluated for estimation of body measurements on pigs in farm conditions, achieving high *R*^2^ (0.77–0.93) between the manual measurement and the measures estimated from the images. Nevertheless, these previous studies used some level of manual handling of the images and they did not evaluate model performance using an independent set of animals or a cross-validation approach. This hampers the evaluation of how generalizable the prediction models are, that is, how these CVS based on 3D cameras would perform in practice. Another drawback of these previous studies is the lack of automation for application in farm conditions, where it would be extremely difficult to manually process the images.

#### Automated Prediction of Individual Body Measurements

Automated non-contact prediction of body weight and body measurements is a long-desired application for many animal production systems. Kashiha et al. (95) developed a CVS for automated prediction of BW in pigs under experimental conditions achieving good prediction (*R*^2^ = 0.97) for body weight using surveillance cameras. However, as stated by the authors, this previous method was still restricted by background and light conditions, along with animal coat color. Recently, Fernandes et al. (14) developed an automated CVS based on depth camera for real-time video processing and prediction of body weight in live pigs. They worked with videos collected under farm conditions using multiple linear regression models with features extracted from the images as predictor variables, achieving high predictive accuracy evaluated with cross-validation (*R*^2^ = 0.92). An adaptation of their CVS was also evaluated for prediction of body weight in beef cattle from depth images (73) achieving high *R*^2^ (0.79–0.91) with an artificial neural network approach. In both works, the images were collected from animals partially restrained and there was only one animal in the camera field of view so that future developments with the CVS on barn conditions would be necessary for better evaluation.

There are also many attempts to develop CVS for automated prediction of body weight and body measurements in fish, where the main challenges are related to fish body positioning and segmentation, and external factors such as light and background conditions. To tackle these issues, one study in halibut developed a CVS based on multi-scale body contour matching and completion using a double local threshold model with body shape priors (80). The final model developed was able to estimate the fish body with an average intersection over union (IoU) of 95.6%. In another study in Nile tilapia, Fernandes et al. (81) used a deep learning approach for fish body segmentation from images under different lighting and background conditions. The approach was able to distinguish fish from the background with a validation IoU of 99%, and for the fish body from fins and background of 0.91%. In their study, the final fish body area was then used for prediction of fish body weight, achieving a predictive *R*^2^ of 0.96. Nevertheless, in this study, the fish were removed from the water, while in the previous one images from the top view of fish in a shallow water area were used. Hence, it is still necessary to develop CVSs that can evaluate fish underwater inside production cages. This adds more challenges, such as interference and occlusion due to different water transparency.

### Perspectives of CVS for High-Throughput Phenotyping

CVS together with other sensor technologies are at the forefront of precision livestock farming, with some systems already been implemented in farm applications (104). Such systems have the potential to enable high-throughput phenotyping (HTP), which can be defined as the measurement of a single or many different traits of interest at multiple times during the animal life. HTP applications promise the generation of large amounts of data that will improve the accuracy of current methods and open a myriad of opportunities to advance breeding programs and livestock production (105). Nevertheless, for their implementation on breeding programs, there is the need to develop automated and robust CVS that are capable of collecting, processing, analyzing, and transmitting individual animal data. For this to happen, several key resources and tools must be developed such as improvement of rural broadbands, data integration, data mining, and novel predictive tools among others (106). A specific strategy used to circumvent the issue of individual animal identification is by using other technologies such as RFID tags, associated with the CVS (72). Nevertheless, there is still room for more progress in the use of image analysis for animal identification. Recent developments in machine learning algorithms for image analysis such as deep learning have shown promising results in other areas such as human face recognition, disease detection, and classification, among others (22). Generally, these algorithms demand very large datasets to be trained such as the Microsoft Common Objects in Context (COCO) (107). Nonetheless, with techniques such as transfer learning of pre-trained models, we expect that deep learning may play an important role in the future development of CVS applications for animal production.

Animal phenotyping, or rather, the measurement of traits of interest, has long been a constant and important practice in animal management and also for the development of breeding programs for different animal production systems. In this manuscript, we discussed CVS as an interesting tool for the collection of such phenotypes without direct interaction with the animals. Thus, in the last decade, several efforts have been made toward the measurement of group-level traits, such as group growth, activity, drinking and feeding behavior, and animal spatial distribution among others with most of the successful applications based on standard digital cameras implementing classic image analysis and machine learning algorithms. Nevertheless, most of the works in the literature deals with a small group of animals, with just a few works evaluating CVS in farm environments (14, 66, 67, 97) or under challenging light and background conditions (80, 81, 90) with the application of more sophisticated machine learning algorithms.

Nevertheless, there are already some examples of how CVS can be leveraged by breeding programs. In a study by Moore et al. (108), data from 17,765 image carcass records of prime cuts and carcass weight of commercial beef slaughter was used to predict genetic parameters in beef cattle. The authors concluded that by leveraging the information from the CVS it was possible to yield more accurate genetic parameters due to the higher volume of data. In another study, Nye et al. (74) developed a web scraper and an image segmentation algorithm to extract images and information from breeding programs catalogs. The information retrieved was used in a subsequent step to predict genetic parameters related to coat pigmentation and conformation traits in dairy cattle. The authors demonstrated that, for dairy cattle, approximately only 50 images were required to train their semi-supervised machine learning approach.

## Concluding Remarks

The idea of developing CVSs for automatic monitoring and measuring traits of interest in animals is not new. Early developments in digital image analysis and computer vision have shown the potential of the use of images to evaluate animal behavior, gait, body weight, and other traits in experimental conditions, with some more recent studies evaluating also on-farm applications. Also, there are studies showing that different imaging technologies can be better suited for specific applications, such as IRT for identification of mastitis and digital dermatitis in dairy cattle, or spectral and hyperspectral imaging in food sciences. However, there is also a great number of attempts to develop CVS based on more accessible technologies such as standard digital cameras and 3D cameras.

Applications of CVS in animal and veterinary sciences are currently a growing research area. Even though there are already some commercial products for monitoring groups of live animals, or slaughtered animals at the abattoir, there are still several challenges that demand intense research for the successful development and deployment of practical solutions. Current challenges involve the development and implementation of reliable CVS for the autonomous acquisition of data regarding single or multiple traits in farm conditions, as there are still few studies that evaluated these CVS using validation data sets, including different animals in the same farm or across multiple farms. Another area of importance is individual animal identification and tracking since most of the currently developed methods are still prone to error. There is also the need for the development of methods to connect the increasing number of devices used for different applications. This may enable the implementation of more sophisticated predictive algorithms based on multiple inputs and multiple outputs (joint prediction of multiple traits). Finally, there is the need for the development of applications for the delivery of the information generated by the CVS to connected systems thus generating valuable information to farmers and managers. This is the focus of areas such as big data and internet of things which, even though are not the focus of this review, these areas are going to be indispensable for the further development of CVS animal breeding programs and production systems.

## Author Contributions

AF, JD, and GR contributed to the conception and design of the review. AF wrote the first draft of the manuscript. All authors contributed to manuscript revision, read, and approved the submitted version.

## Conflict of Interest

The authors declare that the research was conducted in the absence of any commercial or financial relationships that could be construed as a potential conflict of interest.
